# Announcing the 2017 *Toxins* Travel Awards for Post-Doctoral Fellows and Ph.D. Students

**DOI:** 10.3390/toxins9010037

**Published:** 2017-01-17

**Authors:** Vernon L. Tesh

**Affiliations:** Department of Microbial and Molecular Pathogenesis, Medical Research and Education Building, Room 3002, College of Medicine, Texas A&M University System Health Science Center, 8447 State Highway 47, Bryan, TX 77807, USA; tesh@medicine.tamhsc.edu

As Editor-in-Chief of *Toxins*, I am pleased to announce the winners of the *Toxins* Travel Awards for 2017. We had originally planned to fund two awards, but the overall quality of the applications was stellar, and we couldn’t narrow down the winners to just two! We sought additional funding from leadership at MDPI, and I am delighted that they honored our request to fund three *Toxins* Travel Awards this year.

Travel Awards were granted to: Dr. Marco Pirazzini, a postdoctoral researcher in Dr. Cesare Montecucco’s lab in the Department of Biomedical Sciences, University of Padova, Italy; Dr. Natalie Saez, a postdoctoral researcher in Dr. Glenn King’s lab in the Institute for Molecular Bioscience, University of Queensland, Australia; and Ms. Rachel A. Miller, Ph.D. student in Dr. Martin Wiedmann’s lab, Department of Food Science, Cornell University, USA (Figure 1). 

Dr. Marco Pirazzini has worked in Dr. Cesare Montecucco’s lab, at the University of Padova first as a Ph.D. student and then as a Postdoc. He received his Ph.D. in Biosciences and Biotechnology: Cell Biology in 2013. Under the supervision of Dr. Cesare Montecucco, his focus of research, starting from the PhD, is the study of the molecular mechanism of action of botulinal neurotoxins (BoNTs) expressed by *Clostridium botulinum*. In particular, Dr. Pirazzini is interested in intracellular routing and processing of BoNTs, including the translocation of the catalytic subunit within the cytosol and the reduction of the interchain disulphide bond. His work has important implications for the development of inhibitors of BoNTs. Dr. Pirazzini has co-authored 19 papers, including 7 first author publications. He has published in such high impact journals as *Nature Reviews of Microbiology* and *Cell Reports*. Dr. Pirazzini will use his *Toxins* Travel Grant to present work at TOXINS2017, sponsored by the International Neurotoxin Association in Madrid, Spain, January 18-21, 2017.

Dr. Natalie Saez received her Ph.D from University of Queensland, Australia in 2013, where she worked in the laboratory of Dr. Glenn F. King. Her Thesis involved a detailed study of the molecular basis by which the tarantula toxin PcTx1 inhibitors acid-sensing ion channel 1a. Upon completion of her PhD, she joined the European VENOMICS Project, was involved in the recombinant production of animal venom peptides for the research and development phase of this project. Dr. Saez returned to Dr. Glenn King’s lab after completion of the project, she continued to pursue her interest in the exploration of animal venoms for the discovery of new therapeutics. Her work focuses on understanding the interactions of toxins with ion channels, in particular acid-sensing ion channels and voltage-gated sodium channels. In Dr. Glenn F. King’s letter of support, he wrote “I believe that Dr. Saez is an extremely talented young scientist with a very bright future in the field of toxinology“. Dr. Saez has co-authored 11 peer-reviewed papers. Her review in *Toxins* entitled “Spider-venom peptides as therapeutics” is one of the 10 most highly cited articles in the journal. Dr. Saez will attend the 19th International Society on Toxinology World Congress on Animal, Plant and Microbial Toxins, Hainan, China, 24–30 October 2017.

Ms. Rachel A. Miller started her Ph.D. in 2012, in Dr. Martin Wiedmann’s laboratory, where her research focuses on the role of the cytolethal distending toxin (CDT) in the pathogenesis of nontyphoidal *Salmonella* infections. She also studies the distribution, regulation, and contributions to pathogenicity, of the toxins that are produced by *Bacillus cereus* group species. Ms. Miller’s Ph.D. advisor, Dr. Martin Wiedmann, writes: “Rachel is an outstanding student and constantly challenges herself in every aspect of her academic life.” Ms. Miller has co-authored 8 papers, including 4 as first author. She will present her work at ASM Microbe (joint meeting of ASM and ICAAC) in New Orleans, Louisiana, 1–5 June 2017.

The editors, managing editor and editorial board members congratulate Dr. Pirazzini, Dr. Saez and Ms. Miller on winning 2017 *Toxins* Travel Awards. We are grateful to all who submitted applications—thank you for letting us get to know you and your work. The future of toxinology looks very bright indeed. Once again, we are grateful to MDPI for their generous support of young scholars, helping to share their work on an international stage.

## Figures and Tables

**Figure 1:**
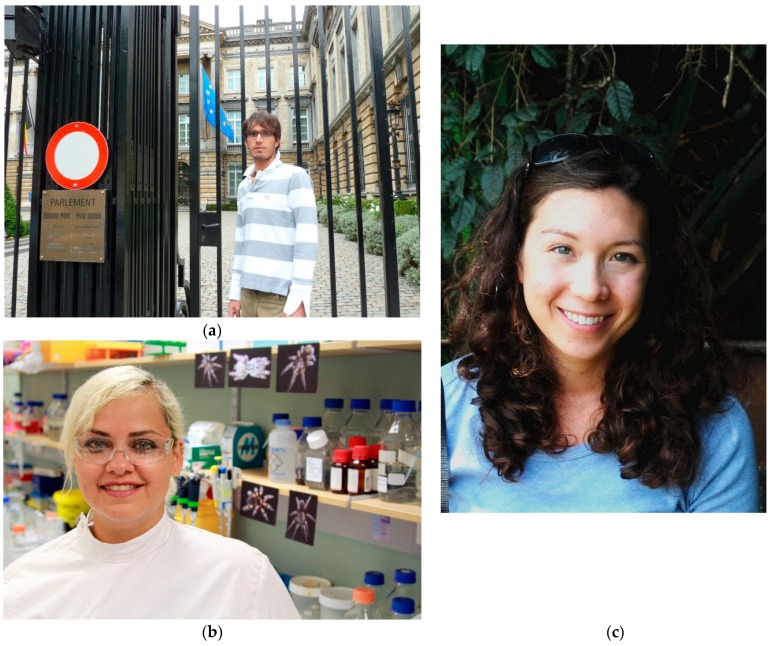
(**a**) Dr. Marco Pirazzini; (**b**) Dr. Natalie Saez; (**c**) Ms. Rachel A. Miller.

